# Maternal lipid profiles in women with and without gestational diabetes mellitus

**DOI:** 10.1097/MD.0000000000015320

**Published:** 2019-04-19

**Authors:** Jing Wang, Zhi Li, Li Lin

**Affiliations:** Department of Obstetrics and Gynecology, Peking University International Hospital, Beijing, China.

**Keywords:** gestational diabetes mellitus, lipid profiles, triglycerides to high-density lipoprotein ratio

## Abstract

To determine the longitude lipid profiles in women with and without gestational diabetes mellitus (GDM), and to investigate the relationship between lipid disturbances in the 1st trimester and GDM.

Blood samples were collected from 1283 normal pregnant women and 300 women with GDM. Serum lipids which include total cholesterol (TC), triglycerides (TGs), low-density lipoprotein cholesterol (LDL-C), and high-density lipoprotein cholesterol (HDL-C) were measured and the TG/HDL-C ratio was calculated in the 1st, 2nd, and 3rd trimesters of pregnancy and then we got the longitudinal lipid profiles. We compared the differences of lipid profiles between patients with GDM and normal pregnant women using 2-way repeated measures analysis of variance. Also additional propensity-based subgroup analyses were performed. The logistic regression analysis was used to determine the relationship between the lipid disturbances in the 1st trimester and GDM.

TG, TC, LDL-C concentrations, and TG/HDL-C ratio increased progressively throughout pregnancy; while HDL-C amounts increased from the 1st to the 2nd trimester with a slight decrease in the 3rd trimester. The GDM group showed higher TG concentrations, higher TG/HDL-C ratio, and lower HDL-C concentrations throughout pregnancy. There were no significant differences in TC and LDL-C concentrations in the 1st, 2nd, and 3rd trimesters (*P* > .05), between the GDM group and the control group. Logistic regression analysis showed that maternal age, prepregnancy body mass index (BMI), and TG/HDL ratio in the 1st trimester were associated with an increased risk of GDM.

The lipid profile alters significantly in patients with GDM, and maternal age, prepregnancy BMI, and TG/HDL ratio in the 1st trimester were associated with an increased risk of GDM.

## Abstract 2

Supplemental Digital Content is available in the text

## 1 Introduction

Gestational diabetes mellitus (GDM), defined as abnormal glucose tolerance firstly discovered during pregnancy, is the most common metabolic disease during pregnancy.^[1]^ Its incidence is increasing worldwide and 15% to 22% of all pregnancies are affected. Due to the new diagnostic criteria, this prevalence may be higher.^[2]^ GDM can cause many consequences, such as fetal macrosomia, preeclampsia and high cesarean section rate, and so on.^[3,4]^ Also both women with GDM and their offspring experience a greater risk of obesity, type 2 diabetes, and cardiovascular disorders in the future.^[5]^

Maternal dyslipidemia which elevated over a physiologic range evidently is a common phenomenon during pregnancy.^[6]^ Especially, hyperlipidemia is commonly discovered in the 2nd half of pregnancy, and it is regarded as a physiologically required mechanism to provide fuel and nutrients for the fetus.^[7]^ The lipid level increases slightly in early pregnancy, but significantly in later pregnancy. However, to ascertain which level of lipid elevation is physiologic or pathologic is difficult nowadays.

The GDM is associated with increased lipid profiles. Although the literature describing lipid levels in pregnancy and GDM is extensive, results have been inconsistent.^[8–11]^ Also there are few studies as to whether lipid patterns differ in women with GDM early in pregnancy and whether lipid abnormalities in the 1st trimester have potential clinical utility for identifying women at risk for subsequently developing GDM.^[12]^ In this study, we sought to perform an observational study to compare the concentrations of total cholesterol (TC), triglycerides (TGs), low-density lipoprotein cholesterol (LDL-C), high-density lipoprotein cholesterol (HDL-C), and TG/HDL-C ratio (TG to HDL-C ratio) in the 1st, 2nd, and 3rd trimesters of pregnancy in normal pregnant women and women with GDM. We aim to determine the longitudinal lipid levels by trimester and compare the differences between patients with GDM and normal pregnant women. Also we aim to determine whether lipid disturbances early in the 1st trimester of pregnancy are related to GDM.

## 2 Materials and methods

### 2.1 Setting and participants

This retrospective cohort study was conducted at Perking University International Hospital (PKUIH). This study was reviewed and approved by the ethics review board of PKUIH and the institutional review board approval number was 2018-020. A total of 1583 women who attended regular prenatal health care and delivered their babies in PKUIH from January to December 2017 were included in this study. Among them, 300 pregnancy women who were diagnosed as GDM in the 2nd trimester according to the American Diabetes Association (ADA) 2010 criteria^[13]^ were classified as patients with GDM, while 1283 healthy pregnant women were included in the control group.

Inclusion criteria of pregnant women were: singleton pregnancy; had integrated medical records. Exclusion criteria of pregnant women were: multiple pregnancies; type 1 or type 2 diabetes mellitus before pregnancy, inherited metabolic diseases, cardiovascular disease, thyroid or liver dysfunction before pregnancy; use of drugs that could affect lipid levels, including corticosteroids, etc.

The information of maternal age, height, parity, prepregnancy body mass index (BMI), delivery mode, gestational age, birth weight, etc, were recorded. Prepregnancy BMI was derived as the weight (kg) divided by the square of the height (m), and the patients were classified as low weight (<18.5 kg/m^2^), normal weight (18.5–24.9 kg/m^2^), overweight (25.0–29.9 kg/m^2^), or obese (≥30.0 kg/m^2^) on the basis of World Health Organization BMI classification.^[14]^ Macrosomia is defined as birth weight ≥4000 g.

### 2.2 Biochemical analyses

Longitudinal lipid assessments were carried out during three periods: 6 to 8 gestational weeks (GWs) (the 1st trimester, T1), 24 to 28 GWs (the 2nd trimester, T2), and 32 to 34 GWs (the 3rd trimester, T3). TG/HDL-C ratio was calculated accordingly by trimester.

Blood samples were collected at the outpatient clinic by a trained nurse after a 10- to 12-hour fasting period. Serum TC, LDL-C, HDL-C, TG, and glucose concentrations were measured on an automatic biochemical analyzer (Beckman Coulter Co. Ltd., Tokyo, Japan) and monitored by a well-trained inspector. The inter- and intra-assay coefficients of variation were <1.6%, 0.6% (TC); <1.7%, 1.1% (TG); <1.1%, 0.6% (HDL-C); and <1.6%, 1.1% (LDL-C), respectively.

### 2.3 Statistical analysis

In our study, descriptive statistics included means and standard deviation (SD) for continuous variables, and numbers and percentages for categorical variables. First, we used 2-way repeated measures analysis of variance to estimate whether serum TC, TG, LDL-C, HDL-C concentrations, and TG/HDL-C ratio increased by trimesters (*P* for trend) and explore the differences in serum lipids concentrations in the 1st, 2nd, and 3rd trimesters between pregnant women with and without GDM (*P* for difference). Then, we used backward stepwise logistic regression analysis to test whether serum lipids measured in the 1st trimester could predict the risk of GDM in the 2nd trimester, and the candidate predictor variables included maternal age, prepregnancy BMI, and parity as confounding variables. Finally, to test robustness of our main results, we performed additional propensity-based subgroup analyses. Pregnant women with and without GDM were matched (1:1) on maternal age, prepregnancy BMI, and parity, using the Greedy matching macro.^[15]^

All the analyses were performed with SPSS version 20.0 for Windows (SPSS Inc, Chicago, IL). *P* values <.05 were defined as statistically significant.

## 3 Results

### 3.1 Characteristics of the study population

Table 1 presents maternal and neonatal characteristics of our study population. Among the 1583 mothers in the present study, the maternal age in the GDM group was 32.65 ± 3.92 years, while that of the control group was 31.53 ± 3.68 years, with significantly statistical difference between the 2 groups. The prepregnancy BMI in the GDM group was 23.22 ± 3.49 kg/m^2^, which was significantly higher than that of the control group (21.87 ± 2.97 kg/m^2^), *P* < .001. The mean age at delivery was 39.20 ± 1.01 weeks in the GDM group and 39.43 ± 1.21 weeks in the control group, with statistical difference between the 2 groups. There were no significantly statistical differences on the birth weight, the rate of primiparous, the rate of caesarean section, and the rate of macrosomia between the 2 groups.

**Table 1:**
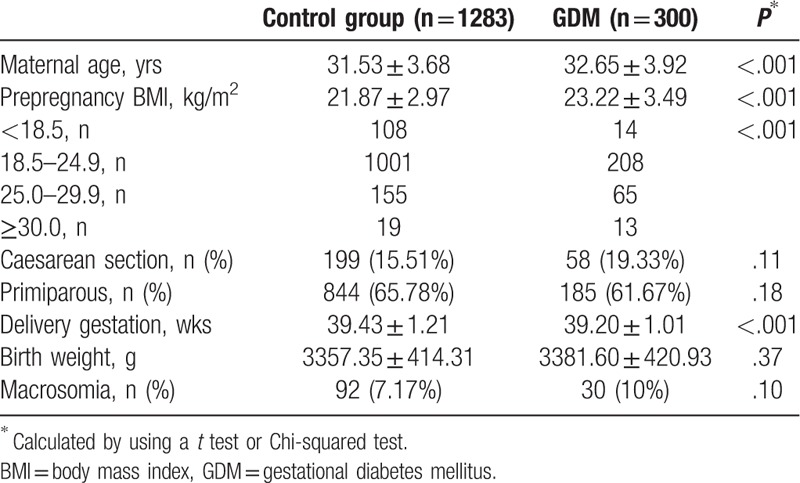
Characteristics of study population.

### 3.2 Maternal lipid profiles by trimester

Table 2 shows maternal lipid profiles by trimester. Serum TG, TC, LDL-C concentrations, and TG/HDL-C ratio increased progressively throughout pregnancy (all *P* for trend <.001). However, HDL-C amounts increased from the 1st to the 2nd trimester with a slight decrease in the 3rd trimester. All the lipid parameters of the 3rd trimester were higher than those of the 1st and 2nd trimesters, except HDL-C. Similarly, the levels of lipids increased by increasing trimesters in both GDM and control groups (all *P* for trend <.001, Supplement Table 1).

**Table 2:**
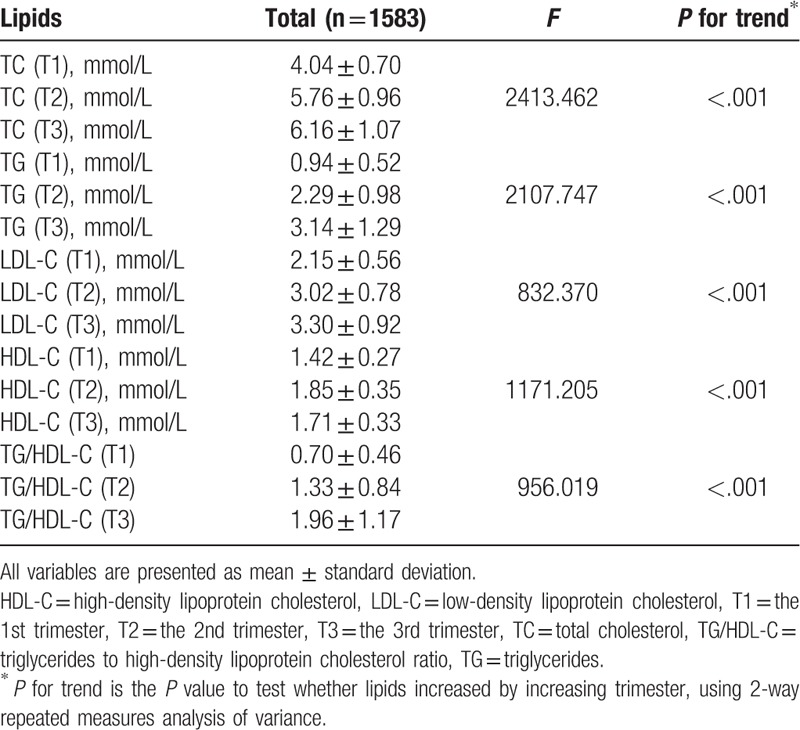
Maternal lipid profiles by trimester.

### 3.3 Maternal lipid profiles between the 2 groups

**Figure 1:**
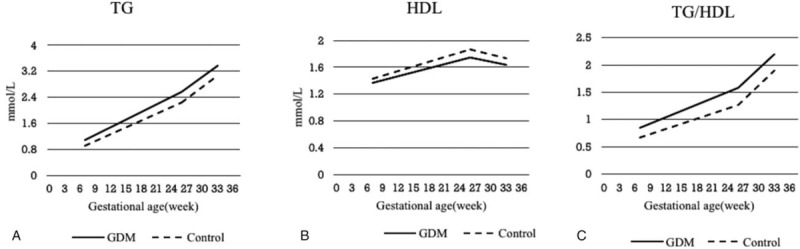
Unadjusted values of lipids (TG, HDL-C, and TG/HDL-C ratio) in the 2 groups in different trimesters. GDM = gestational diabetes mellitus, HDL-C = high-density lipoprotein cholesterol, TG = triglycerides, TG/HDL-C = triglycerides to high-density lipoprotein cholesterol ratio.

Compared with the control group, the GDM group showed higher TG concentrations and higher TG/HDL-C ratio throughout pregnancy, while lower HDL-C concentrations throughout pregnancy (*P* < .05). However, there were no significant differences in TC and LDL-C concentrations in the 1st, 2nd, and 3rd trimesters between the GDM group and the control group (*P* > .05) (Table 3). Unadjusted values of lipids (TG, HDL-C, and TG/HDL-C ratio) in the 2 groups in different trimesters are depicted in Figure 1.

**Table 3:**
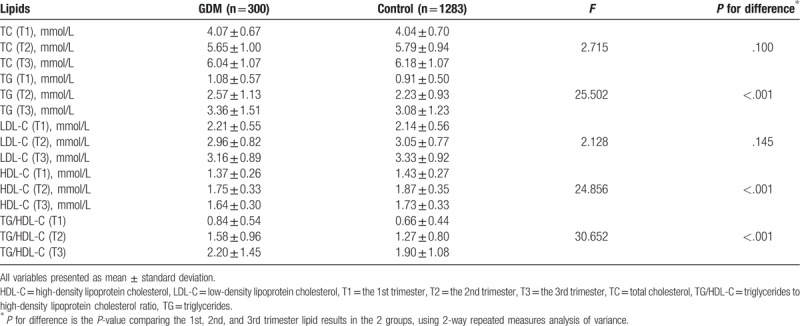
Maternal lipid profiles of the different trimesters in the 2 groups.

### 3.4 Longitudinal analysis

Logistic regression analysis showed that maternal age, prepregnancy BMI, and TG/HDL ratio measured in the 1st trimester were associated with an increased risk of GDM (Table 4).

**Table 4:**
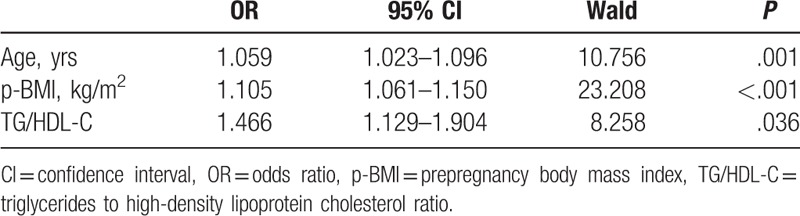
Binary logistic regression analysis of the risk of GDM.

### 3.5 Propensity score analysis

The propensity-score matching identified 297 pregnant women with GDM and 297 pregnant without GDM. Maternal age, prepregnancy BMI, and parity were comparable between 2 groups (see Supplement Table 2). Similar to the main analysis, these analyses showed that TG, TC, LDL-C concentrations, and TG/HDL-C ratio increased progressively throughout pregnancy; while HDL-C amounts increased from the 1st to the 2nd trimester with a slight decrease in the 3rd trimester. Compared with the control group, the GDM group showed higher TG/HDL-C ratio and lower HDL-C concentrations throughout pregnancy, with no significant difference on TG concentrations. The difference of TG concentration was no longer significant probably because of the relatively small sample size (Supplement Tables 3–4).

## 4 Discussion

Lipid metabolism is essential for a healthy pregnancy development.^[16]^ The plasma lipid profile including the levels of TC, HDL-C, LDL-C, and TG changes apparently during normal pregnancy.^[17,18]^ Plasma lipid concentrations increase markedly during pregnancy due to estrogen stimulation and insulin resistance.^[19]^ During the 1st two-thirds of gestation, there is an increase in maternal fat accumulation, associated with both hyperphagia and increased lipogenesis.^[20–22]^ In the last 3rd trimester of gestation, however, as a result of increased lipolytic activity and declined lipoprotein lipase activity, maternal fat storage decreases or even ceases.^[23,24]^ These changes are reflections of maternal physiologic adaptation to energy demand of the fetus, also they are necessary preparations for delivery and lactation.^[16]^

Circulating plasma lipid patterns during normal pregnancy have been widely studied, and most studies have found that serum TG, HDL-C, LDL-C, and TC levels are obviously elevated throughout the pregnancy.^[25–27]^ Shen et al found that the levels of lipids, including TG, TC, and LDL-C, increased gradually during gestation and peaked before delivery; meanwhile, HDL-C amounts increased from the 1st to 2nd trimester with a slight decrease in the 3rd trimester.^[8]^ Consistent with Shen's study, we also found the similar lipid profiles throughout the pregnancy. And this indicated that the elevation of lipid concentrations is a physiologic requirement for maintaining stable energy storage for the fetus. However, it is difficult to ascertain which level of lipid elevation is physiologic or pathologic and there are no worldwide standard criteria of lipid levels during pregnancy due to the heterogeneity of the population and territory.

Hypertriglyceridemia and low HDL-C levels are 2 important metabolic abnormalities associated with insulin resistance.^[28,29]^ Moreover, the TG/HDL-C ratio was proved to be a good and sensitive indicator to identify insulin-resistant individuals of North American aboriginal, Chinese, and European.^[29]^ The TG/HDL-C ratio was considered as an atherogenic index.^[30]^ In our study, we found that the TG/HDL-C ratio increased significantly throughout the pregnancy, which indicated progressive insulin resistance during normal pregnancy.

The GDM is a complex condition that manifests as glucose intolerance and insulin resistance with onset or 1st recognition during pregnancy.^[31]^ Women with GDM are at highly increased risk of developing metabolic diseases after pregnancy including hyperlipidemia, type 2 diabetes, and so on.^[32,33]^

There have been extensive studies about circulating lipid patterns in GDM vs normal pregnancy, with no consistent conclusions.^[34]^ Except for a few studies in which no changes of serum TG levels were found in GDM group compared to nondiabetic group,^[35–37]^ most studies suggested that women with GDM have increased levels of TG, LDL-C, and TC and lower levels of HDL-C. Savvidou et al found that women who developed GDM had higher TG, TC, LDL-C levels, and lower levels of HDL-C in univariate analysis.^[38,39]^ Shen et al^[8]^ found that high levels of TGs during pregnancy were associated with increased risk of GDM and gestational weight gain during gestation; TC and LDL-C levels were only higher in the 1st trimester for the GDM group, with no difference of HDL-C levels. A recent meta-analysis^[9]^ showed that serum TG was significantly elevated among GDM women and the increase persisted across all the 3 trimesters of pregnancy. They also found that serum HDL-C levels were significantly lower in women with GDM in the 2nd and 3rd trimesters of pregnancy compared with women without insulin resistance. However, no elevated serum TC and LDL-C levels were found between women with GDM and women without insulin resistance in the study. Among the Chinese population, Jin et al^[40]^ found that maternal high TG in late pregnancy was independently associated with increased risk of GDM; and relatively lower maternal HDL-C during pregnancy had a significant association with increased risk of GDM and macrosomia; indicating that high HDL-C was a protective factor for both of them. In a recent study, Khosrowbeygi et al found the atherogenic indexes including the LDL-C/HDL-C, TG/HDL-C, and TC/HDL-C ratios were higher in GDM compared with normal pregnancy and especially, the TG/HDL-C ratio showed a significantly positive correlation with insulin resistance, which might be used as a simple surrogate marker for assessing insulin resistance in pregnancy.^[30]^ Similar to the results of the recent meta-analysis,^[9]^ in our study, we found that compared with the control group, the GDM group showed higher TG concentrations, TG/HDL-C ratio, and lower HDL-C concentrations throughout pregnancy. In the matched-pairs analysis, after adjusting age and prepregnancy BMI, we found significant difference on HDL-C concentrations, but no significant difference on TG concentrations between the 2 groups. The phenomena may be probably because of the relatively small sample size, or because serum TG had a closer correlation with age and prepregnancy BMI, and serum HDL-C concentrations maybe more related to GDM than serum TG. But further studies with lager sample size in matched-pairs analysis will be needed.

This study indicated that lipid profiles were dramatically different between the GDM group and the control group and GDM women had more serious dyslipidemia and insulin resistance in pregnancy. Meanwhile, we found that there were no significant differences in TC and LDL-C concentrations in the 1st, 2nd, and 3rd trimesters between the 2 groups. However, the exact mechanism was unknown and further studies should be conducted to explore the role of dyslipidemia in the pathogenesis of GDM.

To examine whether lipid abnormalities in the 1st trimester have potential clinical utility for identifying women at risk for subsequently developing GDM, we analyzed the relationship between maternal lipid profile in the 1st trimester and GDM. Logistic regression analysis showed that maternal age, prepregnancy BMI, and TG/HDL ratio were associated with an increased risk of GDM. This indicated that TG/HDL-C ratio in the 1st trimester in combination with maternal age and prepregnancy BMI could be good markers to predict the risks of GDM. Similarly, Wang et al^[41]^ found that TG/HDL-C ratio in combination with HbA1c and prepregnancy BMI were good markers to predict the risk of GDM and delivering large for gestational age infant. Therefore, the TG/HDL-C ratio might be useful for assessing insulin resistance in pregnancy, and its level in the early pregnancy may be a good predictor for occurrence of GDM. So, in clinical practice, elderly primipara should be reduced, and overweight or obese women are suggested to reduce weight to normal weight before pregnancy. Special attention should be paid to women with abnormal higher TG/HDL ratio in the 1st trimester, and obstetricians should inform them of necessary diet management, weight control, and proper exercise according to 2016 WHO recommendations on antenatal care.^[42]^

In the future, more research is needed to establish the lipid standard during pregnancy according to local maternal characteristics such as inheritance, ethnicity, region, etc. More studies will be needed to explore the correlation between dyslipidemia and the pathogenesis of GDM. Also for women with elderly maternal age, high prepregnancy BMI, elevated TG/HDL-C ratio in the 1st trimester, close surveillance and monitoring, necessary weight control, and diet management should be carried out to reduce the incidence of GDM as much as possible.

### 4.1 Main findings

This study yielded 3 main findings: maternal TG, TC, LDL-C concentrations, and TG/HDL-C ratio increased progressively throughout pregnancy; and HDL-C concentrations increased from the 1st to the 2nd trimester with a slight decrease in the 3rd trimester; Lipid profiles were dramatically different between the GDM group and the control group. Compared with the control group, the GDM group showed higher TG concentrations, TG/HDL-C ratio, and lower HDL-C concentrations throughout pregnancy. However, there were no significant differences in TC and LDL-C concentrations in the 1st, 2nd, and 3rd trimesters, between the GDM group and the control group; Maternal age, prepregnancy BMI, and TG/HDL ratio in the 1st trimester were associated with an increased risk of GDM and could predict the risk of GDM at an early stage.

### 4.2 Strengths and limitations

This was a longitudinal cohort study with large sample size. In this study, serum lipid levels were tested in 3 different trimesters. Although there were several studies about lipid profile during pregnancy, few studies have tested serum lipids longitudinally at multiple points during pregnancy and previous studies were usually small sample size. Moreover, this study performed additional propensity-based subgroup analyses to adjust prepregnancy age and BMI to avoid the influences on serum lipids concentrations in the 2 groups.

However, the present study still has some limitations. First of all, despite relatively large sample size, this study is a single-center study, and lacked population diversity. A multi-center study with different population and regions may be more comprehensive. Secondly, due to ethical reasons, all GDM women received dietary guidance and exercise management once the diagnosis was established, which may affect the lipid levels. Finally, the information of gestational weight gain was not collected, and we failed to get the relationship between gestational weight gain and the lipid changes during pregnancy.

## 5 Conclusion

Overall, in a large longitudinal cohort study, we found that TG, TC, LDL-C concentrations, and TG/HDL-C ratio increased progressively throughout pregnancy; meanwhile, HDL-C amounts increased from the 1st to the 2nd trimester with a slight decrease in the 3rd trimester. Lipid profiles were dramatically different between the GDM group and the control group, except of serum TC and LDL-C concentrations. Maternal age, prepregnancy BMI and TG/HDL ratio in the 1st trimester could predict the risk of GDM at an early stage.

## Acknowledgments

The authors thank Liyuan Tao (Research Center of Clinical Epidemiology, Peking University Third Hospital) and Wuxiang Xie (Peking University Clinical Research Institute) for his their statistical assistance and advice.

## Author contributions

**Conceptualization:** Li Lin.

**Data curation:** Jing Wang.

**Methodology:** Jing Wang, Zhi Li.

**Writing – original draft:** Jing Wang.

**Writing – review & editing:** Zhi Li.

jing WANG orcid: 0000-0002-1074-4279.

## Supplementary Material

Supplemental Digital Content
