# Supplementary material for: Maternal lipid profiles in women with and without gestational diabetes mellitus
Source: Medicine (Baltimore). 2019 Apr 19;98(16):e15320. doi: 10.1097/MD.0000000000015320 (PMC6494372; doi:10.1097/MD.0000000000015320)
Supplement: Supplemental Digital Content [file medi-98-e15320-s001.docx]

Supplement Table 1 Maternal lipid profiles by trimester in GDM and control groups

| Lipids | GDM (n=300) | P for trend* | Control (n=1283) | P for trend* |
| --- | --- | --- | --- | --- |
| TC(T1) (mmol/L) | 4.07±0.67 | <0.001 | 4.04±0.70 |  |
| TC(T2) (mmol/L) | 5.65±1.00 |  | 5.79±0.94 | <0.001 |
| TC(T3) (mmol/L) | 6.04±1.07 |  | 6.18±1.07 |  |
| TG(T1) (mmol/L) | 1.08±0.57 | <0.001 | 0.91±0.50 |  |
| TG(T2) (mmol/L) | 2.57±1.13 |  | 2.23±0.93 | <0.001 |
| TG(T3) (mmol/L) | 3.36±1.51 |  | 3.08±1.23 |  |
| LDL-C(T1) (mmol/L) | 2.21±0.55 | <0.001 | 2.14±0.56 |  |
| LDL-C(T2) (mmol/L) | 2.96±0.82 |  | 3.05±0.77 | <0.001 |
| LDL-C(T3) (mmol/L) | 3.16±0.89 |  | 3.33±0.92 |  |
| HDL-C(T1) (mmol/L) | 1.37±0.26 | <0.001 | 1.43±0.27 |  |
| HDL-C(T2) (mmol/L) | 1.75±0.33 |  | 1.87±0.35 | <0.001 |
| HDL-C(T3) (mmol/L) | 1.64±0.30 |  | 1.73±0.33 |  |
| TG/HDL-C(T1) | 0.84±0.54 | <0.001 | 0.66±0.44 |  |
| TG/HDL-C(T2) | 1.58±0.96 |  | 1.27±0.80 | <0.001 |
| TG/HDL-C(T3) | 2.20±1.45 |  | 1.90±1.08 |  |

All variables presented as mean ± standard deviation. GDM=gestational diabetes mellitus, TC=total cholesterol, TG=triglycerides, LDL-C =low density lipoprotein cholesterol, HDL-C=high density lipoprotein cholesterol, TG/HDL-C= triglycerides to high density lipoprotein cholesterol ratio, T1=the first trimester, T2=the second trimester, T3=the third trimester.

*P for trend is the p-value to test whether lipids increased by increasing trimester, using one-way ANOVA.

Supplement table 2: Characteristics of the study population after matched-pairs process

|  | Control group (n=297) | GDM (n=297) | P* |
| --- | --- | --- | --- |
| Maternal age (years) | 32.74±3.93 | 32.64±3.94 | 0.761 |
| Pre-pregnancy BMI (kg/m^2^) | 23.17±3.60 | 23.22±3.49 | 0.857 |
| Primiparous (n, %) | 177(59.60%) | 183(61.62%) | 0.557 |

*Calculated by using a t test or chi-square test. GDM=gestational diabetes mellitus, BMI=body mass index.

Supplement table 3: Maternal lipid profiles by trimester after matched-pairs process

| Lipids | Total(n=594) | F | P for trend* |
| --- | --- | --- | --- |
| TC(T1) (mmol/L) | 4.09±0.70 | 1257.339 | <0.001 |
| TC(T2) (mmol/L) | 5.66±1.00 |  |  |
| TC(T3) (mmol/L) | 6.05±1.08 |  |  |
| TG(T1) (mmol/L) | 1.05±0.61 | 1083.534 | <0.001 |
| TG(T2) (mmol/L) | 2.48±1.05 |  |  |
| TG(T3) (mmol/L) | 3.28±1.46 |  |  |
| LDL-C(T1) (mmol/L) | 2.21±0.56 | 373.839 | <0.001 |
| LDL-C(T2) (mmol/L) | 2.96±0.79 |  |  |
| LDL-C(T3) (mmol/L) | 3.18±0.90 |  |  |
| HDL-C(T1) (mmol/L) | 1.40±0.27 | 662.609 | <0.001 |
| HDL-C(T2) (mmol/L) | 1.79±0.35 |  |  |
| HDL-C(T3) (mmol/L) | 1.68±0.33 |  |  |
| TG/HDL-C(T1) | 0.79±0.54 | 441.517 | <0.001 |
| TG/HDL-C(T2) | 1.49±0.87 |  |  |
| TG/HDL-C(T3) | 2.12±1.39 |  |  |

All variables presented as mean ± standard deviation. TC=total cholesterol, TG=triglycerides, LDL-C =low density lipoprotein cholesterol, HDL-C=high density lipoprotein cholesterol, TG/HDL-C= triglycerides to high-density lipoprotein cholesterol ratio, T1=the first trimester, T2=the second trimester, T3=the third trimester.

*P for trend is the p-value to test whether lipids increased by increasing trimester, using two-way repeated measures ANOVA.

Supplement table 4: maternal lipid profiles of the different trimesters in the matched-pairs two groups.

| Lipids | GDM(n=297) | control (n=297) | F | P for difference* |
| --- | --- | --- | --- | --- |
| TC(T1) (mmol/L) | 4.06±0.67 | 4.11±0.73 | 0.149 | 0.700 |
| TC(T2) (mmol/L) | 5.66±1.00 | 5.69±0.97 |  |  |
| TC(T3) (mmol/L) | 6.05±1.07 | 6.17±1.09 |  |  |
| TG(T1) (mmol/L) | 1.07±0.56 | 1.03±0.65 | 2.823 | 0.093 |
| TG(T2) (mmol/L) | 2.57±1.13 | 2.39±0.95 |  |  |
| TG(T3) (mmol/L) | 3.36±1.51 | 3.20±1.39 |  |  |
| LDL-C(T1) (mmol/L) | 2.21±0.55 | 2.20±0.56 | 0.034 | 0.854 |
| LDL-C(T2) (mmol/L) | 2.96±0.82 | 2.97±0.77 |  |  |
| LDL-C(T3) (mmol/L) | 3.17±0.89 | 3.20±0.91 |  |  |
| HDL-C(T1) (mmol/L) | 1.38±0.26 | 1.41±0.28 | 6.44 | 0.011 |
| HDL-C(T2) (mmol/L) | 1.76±0.33 | 1.83±0.37 |  |  |
| HDL-C(T3) (mmol/L) | 1.64±0.30 | 1.70±0.35 |  |  |
| TG/HDL-C(T1) | 0.83±0.53 | 0.76±0.54 | 3.994 | 0.046 |
| TG/HDL-C(T2) | 1.57±0.96 | 1.40±0.76 |  |  |
| TG/HDL-C(T3) | 2.20±1.46 | 2.03±1.32 |  |  |

All variables presented as mean ± standard deviation. TC=total cholesterol, TG=triglycerides, LDL-C =low density lipoprotein cholesterol, HDL-C=high density lipoprotein cholesterol, TG/HDL-C= triglycerides to high-density lipoprotein cholesterol ratio, T1=the first trimester, T2=the second trimester, T3=the third trimester.

*P for difference is the p-value comparing the first, second and third trimester lipid results in the matched-pairs two groups, using two-way repeated measures ANOVA.
